# Mental health related Internet use among psychiatric patients: a cross-sectional analysis

**DOI:** 10.1186/s12888-014-0368-7

**Published:** 2014-12-24

**Authors:** Sophie Kalckreuth, Friederike Trefflich, Christine Rummel-Kluge

**Affiliations:** Department of Psychiatry and Psychotherapy, Faculty of Medicine, Leipzig University, Semmelweißstraße 10, 04103 Leipzig, Germany; Forschungszentrum der Stiftung Deutsche Depressionshilfe, Semmelweißstraße 10, 04103 Leipzig, Germany

**Keywords:** Internet, Psychiatry, Availability, Information seeking, Social media, Internet-based interventions

## Abstract

**Background:**

The Internet is of great importance in today’s health sector, as most Internet users utilize online functions for health related purposes. Concerning the mental health care sector, little data exist about the Internet use of psychiatric patients. It is the scope of this current study to analyze the quantity and pattern of Internet usage among mental health patients.

**Methods:**

Patients from all services of the Department of Psychiatry at a university hospital were surveyed by completing a 29-item questionnaire. The data analysis included evaluation of frequencies, as well as group comparisons.

**Results:**

337 patients participated in the survey, of whom 79.5% were Internet users. Social media was utilized by less than half of the users: social networks (47.8%), forums (19.4%), chats (18.7%), blogs (12.3%). 70.9% used the Internet for mental health related reasons. The contents accessed by the patients included: information on mental disorders (57.8%), information on medication (43.7%), search for mental health services (38.8%), platforms with other patients (19.8%) and platforms with mental health professionals (17.2%).

Differences in the pattern of use between users with low, medium and high frequency of Internet use were statistically significant for all entities of social media (p < 0.01), search for mental health services (p = 0.017) and usage of platforms with mental health professionals (p = 0. 048).

The analysis of differences in Internet use depending on the participants’ type of mental disorder revealed no statistically significant differences, with one exception. Regarding the Internet’s role in mental health care, the participants showed differing opinions: 36.2% believe that the Internet has or may have helped them in coping with their mental disorder, while 38.4% stated the contrary.

**Conclusions:**

Most psychiatric patients are Internet users. Mental health related Internet use is common among patients, mainly for information seeking. The use of social media is generally less frequent. It varies significantly between different user types and was shown to be associated with high frequency of Internet use. The results illustrate the importance of the Internet in mental health related contexts and may contribute to the further development of mental health related online offers.

**Electronic supplementary material:**

The online version of this article (doi:10.1186/s12888-014-0368-7) contains supplementary material, which is available to authorized users.

## Background

The Internet is of great importance in today’s health care sector. The majority of Internet users utilize online functions for health related purposes such as to search for information on medical conditions or medication [1-3]. The mental health (MH) care sector is part of this development [4], as the Internet offers a wide range of options for patients suffering from psychiatric disorders, as well as for mental health professionals [5,6]. In the current mental health related research, several Internet-based therapy programs are being examined in randomized controlled trials, including the German language sphere [7-9]. So far, promising results have been shown for the effective web-based treatment of many common psychiatric disorders [10,11] particularly for depression [12-21] and anxiety disorders [22-24]. The successful usage of these programs requires (1) access to the Internet and (2) the capability and willingness to employ social media and eLearning methods, i.e. the application of digital media for teaching and learning [25]. To date, there has been little research about the specific characteristics of Internet use of psychiatric patients, even though mental disorders are frequent in industrialized countries [26] and therefore are of great relevance for health and health related research. Since psychiatric disorders can be cause and effect of social inequalities [27], a disadvantage of this patient population regarding Internet use can be suspected.

To our knowledge, only few surveys investigating the Internet use of mental health patients exist [28,29]. However, these studies are restricted to certain patient populations (mainly inpatients [28], outpatients only [29]), and the data used date back to 2001 [28] and 2007 [29]. Particularly little data exist about social media, such as social networks, blogs and forums. As the Internet is a dynamic medium, which is rapidly changing, we consider it necessary to evaluate the Internet use of psychiatric patients today in order to gain knowledge about the preconditions for mental health related online options. Therefore, we aim to ascertain the following:

1. Internet usage of mental health patients, particularly in terms of social media
2. Mental health patients’ view on the Internet’s therapeutic options
3. Possible differences in (1) and (2), depending on the frequency of Internet use
4. Possible differences in (1) and (2), depending on a patient’s psychiatric diagnosis

Results may contribute to the further development of web-based mental health treatment options, as they will deliver insight into patients’ interests and needs in this context. This could avoid the unfocused creation of online offers without consideration of the target group’s demand.

## Methods

### Study population

This research was conducted at the Department of Psychiatry and Psychotherapy of the University Hospital Leipzig, Germany. All patients who were currently treated in any of the department’s services (inpatient care, outpatient care, day hospital) were invited to take part in the study. Inclusion criteria were: age ≥ 18 years and submission of an informed consent. The exclusion criteria included insufficient knowledge of German, illiteracy and a cognitive impairment making the completion of the questionnaire impossible.

The study was approved by the Ethical committee of the Medical Faculty of Leipzig University.

### Material

The questionnaire used for this study consists of three sections: Socio-demographic data, general Internet use and mental health related Internet use.

In the first section, nine socio-demographic indicators were surveyed using six multiple-choice questions, one time specification and two open-ended questions. General Internet use was assessed by six questions: two multiple-choice questions, one multiple-choice question with room for adding explanations, two time specifications and one open-ended question. The section “mental health related Internet use” included 14 questions: ten multiple-choice questions, three multiple-choice questions with room for adding explanations and one open-ended question.

The total number of items is thus 29. Sample questions are enclosed as Additional file 1. The full questionnaire is available upon request from the corresponding author.

Questions about general Internet use were adapted from the German ARD/ZDF Online study [30], a representative survey on media use in the German public. Based on this general assessment, mental health related topics and questions were added to the questionnaire. For the patients receiving inpatient care, their data were completed with information obtained by clinicians regarding exact medication, current diagnosis and the severity of a patient’s illness. The quantitative rating of the disease severity was obtained using the Global Assessment of Functioning Scale (GAF) [31] and the Clinical Global Impressions Scale (CGI) [32].

### Procedure

Inpatients and patients in the day hospital were addressed in their respective wards. The invitation to take part in the study was distributed orally in a patient group meeting or through a written notice displayed in the common areas. Outpatients were approached in the waiting area of the outpatient service. The correct understanding of the questionnaire was ensured in two ways: Explanatory phrases and examples were included in the questionnaire in order to clarify specific Internet-related terms. As all questionnaires were completed in the presence of the study staff, further support and explanations could be provided during the data acquisition.

The data were collected from February to July 2013.

### Data analysis

The data were treated confidentially and anonymized before evaluation. For all statistical analysis the statistical software package PASW Statistics 18™ for Windows (IBM, New York, USA) was used.

Frequency of reported weekly Internet use was categorized by thirds as high, medium or low; these three groups were then compared. For the analysis of differences depending on the patients’ diagnosis, groups were defined according to ICD-10 categories. Chi-square tests were performed for univariate significance testing, accepting a p value of ≤ 0.05 as statistically significant. Wherever expected frequencies were <5, the Fisher-Freeman-Halton test was used. The comparison of user type and the variables age, GAF and CGI allowed for the employment of the Kruskal-Wallis test, followed by post-hoc testing using the Mann–Whitney-*U* test. Answers to open-ended questions were sorted and classified in correspondent categories by two independent coders.

In order to keep a straight focus on mental health related Internet use we refrained from a detailed comparison with the German public, which will be published elsewhere.

## Results

### Participation rate

346 patients agreed to participate in the study, signed the informed consent and completed the questionnaire. Nine patients had to be excluded from the analysis due to not meeting the inclusion criteria [age <18 (n = 1); not currently treated at the Department of Psychiatry (n = 3); incapability of completing the questionnaire without help (n = 5)]. The final number of participants was therefore 337, consisting of 108 inpatients, 172 outpatients and 57 patients from the day hospital.

The participation rate was calculated counting the number of patients present at the outpatient clinic and the respective inpatient units at the time of assessment. Although it was not always possible to invite all eligible patients to the survey at all times (e.g. due to patients’ absence from the ward at the time of recruitment visits), we encountered a participation rate of 56.5% at the inpatient clinic, 70.2% at the outpatient clinic and 75% amongst day hospital patients. The overall participation rate was thus 66%.

### Sample characteristics

Sociodemographic characteristics of the sample are shown in Table 1. The three user groups showed statistically significant differences for age (*χ*^2^ = 43.2; df = 2; p < 0.001), marital status (FI = 25.3; df = 6; p < 0.001), educational level (FI = 11.4; df = 6; p = 0.048) and occupation (FI = 27.1; df = 14; p = 0.012). Differences in gender were not statistically significant (*χ*^2^ = 4.75; df = 2; p = 0.93). Post-hoc testing revealed statistically significant differences in the comparison of low and medium Internet use for age (Z = −2.0; p = 0.043), gender (*χ*^2^ = 4.1; p = 0.043) and marital status (FI = 12.5; p = 0.003). In the comparison of medium and high Internet use this was the case for age (Z = −3.1; p = 0.002) and marital status (FI = 7.3; P = 0.042). The analysis of low versus high Internet use reported statistically significant results for age (Z = −6.4; p > 0.001), marital status (FI = 18.3; p > 0.001), educational level (FI = 9.7; p = 0.012) and occupation (FI = 22.4; p = 0.001).

**Table 1:** **Sociodemographic sample characteristics**

		**Entire sample (n = 337)**	**Low internet use (n = 78)**	**Medium internet use (n = 88)**	**High internet use (n = 85)**
**Age mean (±SD)**		46.0 (±16.3)	49.2 (±13.4)	40.9 (±13.7)	35.2 (±13.7)
**Gender %**					
	*Female*	57.3	66.7	51.1	52.9
	*Male*	42.7	33.3	48.9	47.1
**Marital Status %**					
	*Unwed*	43.0	30.8	47.7	63.5
	*Married/living with partner*	32.3	33.3	38.6	21.2
	*Divorced/seperated*	20.5	32.1	13.6	14.1
	*Widowed*	4.2	3.8	0.0	1.2
**Educational level %**					
	*No school degree*	1.2	0.0	1.2	1.2
	*Mandatory school*	55.4	61.0	45.9	47.1
	*High school*	17.8	11.7	23.5	30.6
	*University degree*	25.6	27.3	29.4	21.2
**Occupation %**					
	*Unemployed*	17.2	18.7	21.2	21
	*Apprentice/trainee*	2.8	1.3	4.7	4.9
	*University student*	6.2	0.0	5.9	17.3
	*Employee*	22.8	32.0	25.9	27.2
	*Self-employed*	4.6	4.0	9.4	3.7
	*Housewife/househusband*	2.2	2.7	1.2	2.5
	*Retiree*	37.2	32.0	24.7	14.8
	*Other*	7.1	9.3	7.1	8.6

The patients participating in this research were diagnosed with the following mental disorders: 44.2% (149/337) affective disorders [ICD-10 diagnoses F30-F39]; 17.8% (60/337) schizophrenia [ICD-10 diagnoses F20-F29]; 17.8% (60/337) neurotic, stress-related and somatoform disorders [ICD-10 diagnoses F40-F49]; 8.0% (27/337) organic mental disorders [ICD-10 diagnoses F00-F09]; 5.6% (19/337) disorders of adult personality and behavior [ICD-10 diagnoses F60-F69]; 3.0% (10/337) disorders due to psychoactive substance use [ICD-10 diagnoses F10-F19] and 3.6% (12/337) other disorders [ICD-10 diagnoses F50-F59,F70-F99].

51% of the participants were outpatients, 32% were inpatients and 17% were day hospital patients. The mean GAF score was 56 (SD ± 16), corresponding to the category: “Moderate symptoms and/or moderate difficulty in social, work or school functioning” [31]. For the CGI, a mean score of 4.1 (SD ± 1.1) was calculated, classifying patients as “moderately ill” [32].

### Internet use of mental health patients

#### General Internet use and user definition

79.5% of all participants (268/337) reported having used the Internet at least once, and therefore were classified as *Internet users*. All following analysis refers to the 268 Internet users. Patients were divided into one of three categories according to their reported weekly frequency of Internet use: 3.5 hours or less [*low Internet use* (n = 78)], more than 3.5 and less than 12.5 hours a week [*medium Internet use* (n = 88)], 12.5 hours and more [*high Internet use* (n = 85)]. 17 patients had not provided information on their weekly Internet use and therefore could not be classified.

A negative correlation was found between age and reported frequency of Internet use with a Pearson’s coefficient of r = −0.261 (p < 0.01), showing that older patients used the Internet less frequently than younger patients.

### Usage of social media

Analysis of the responses revealed that 47.8% of the Internet users (128/268) utilized social networks. 19.4% (52/268) took part in online forums, 18.7% (50/268) used web-based chat functions and 12.3% (33/268) read or wrote blogs.

**Figure 1:**
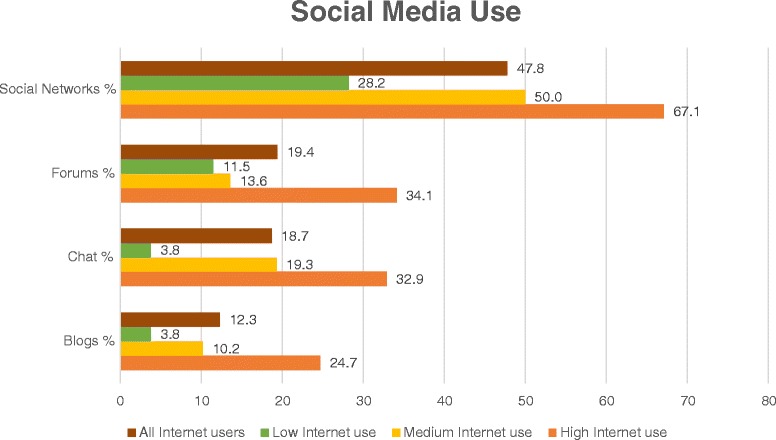
**Social media use by user type.** Patients were asked to indicate their usage of social media (multiple answers possible). The figure illustrates the percentage of use in the sub-samples (low, medium and high Internet use) with the mean of all Internet users as a reference value.

Figure 1 illustrates the differences between the three groups of Internet users, which were statistically significant for all entities of social media: social networks (*χ*^2^ = 24.6; df = 2; p < 0.01), forums (*χ*^2^ = 16.4; df = 2; p < 0.01), chat (*χ*^2^ = 22.3; df = 2; p < 0.01), blogs (*χ*^2^ = 16.5; df = 2; p < 0.01). Further analysis of the subgroups showed the following results: When comparing only low and medium Internet use these differences were statistically significant for the use of social networks (*χ*^2^ = 8.2; df = 1; p = 0.004) and chat (*χ*^2^ = 9.3; df = 1; p = 0.002). Between medium and high Internet use statistically significant differences were shown for social networks (*χ*^2^ = 5.2; df = 1; p = 0.023), forums (*χ*^2^ = 10.0; df = 1; p = 0.002), chat (*χ*^2^ = 4.2; df = 1; p = 0.041) and blogs (*χ*^2^ = 6.3; df = 1; p = 0.012). In the comparison of low and high Internet use statistical significance was revealed for social networks (*χ*^2^ = 24.6; df = 1; p < 0.001), forums (*χ*^2^ = 11.6; df = 1; p = 0.001), chat (*χ*^2^ = 22.4; df = 1; p < 0.001) and blogs (*χ*^2^ = 14.1; df = 1; p < 0.001).

### Mental health related Internet use

**Figure 2:**
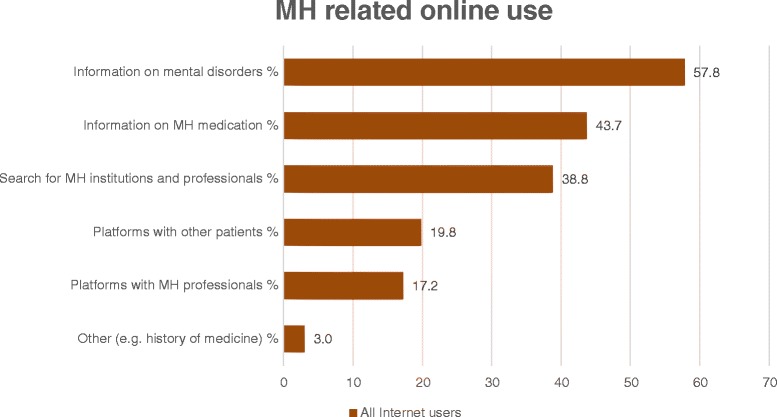
**Mental health related online use.** This question explores the mental health related online contents accessed by the patient (multiple answers possible). Results are displayed as a percentage of all 268 Internet users.

70.9% of the participating patients (190/268) had already used the Internet for mental health related reasons. Figure 2 illustrates the types of information and online contents accessed by the patients.

131 answers were given to an open-ended question about the websites used for mental health related information. 57.3% (75/131) indicated search engines, 19.8% (26/131) cited the online encyclopedia Wikipedia, while 11.5% (15/131) named diagnosis-specific websites. 5.3% (7/131) stated health portals, 3.8% (5/131) indicated forums and 2.3% (3/131) specified hospital websites.

### Online search for and communication with mental health professionals

In our sample, 38.8% (104/268) used the Internet to search for mental health services and mental health professionals. 16.8% (45/268) had established contact with a mental health professional via Internet before, in contrast to 74.6% (200/268) who had never done so. Of those, 66.0% (132/200) believed that the Internet could facilitate the approaching of mental health professionals, whereas 32.0% (62/200) stated the contrary. Regular communication with mental health professionals via Internet was reported by 7.1% (19/268) of our sample.

**Figure 3:**
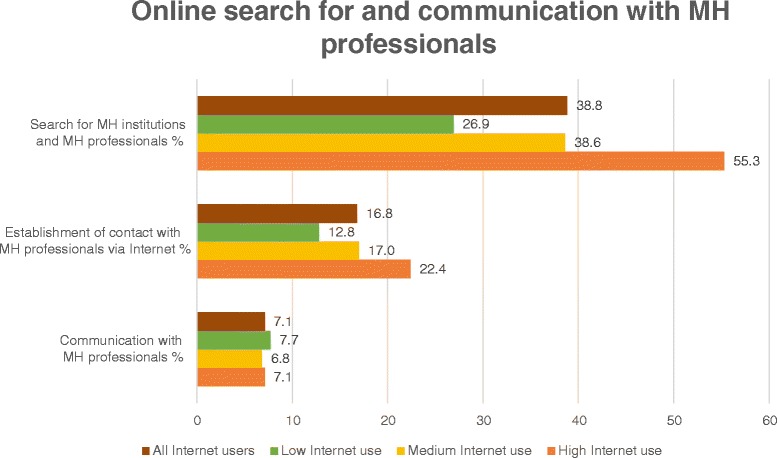
**Online search for and communication with mental health professionals by user type.** This figure illustrates the percentage of Internet users in the subsamples of low, medium and high Internet use, who carried out mental health-specific actions on the Internet. The percentage among all users is a reference value.

Figure 3 shows the frequency of online search for and communication with mental health professionals for low, medium and high Internet use.

Regarding the search for mental health services or mental health professionals, a statistically significant difference between user groups was detected (*χ*^2^ = 8.2; df = 2; p = 0.017). In the comparison of subgroups a statistically significant difference was shown between medium and high Internet use (*χ*^2^ = 4.1; df = 1; p = 0.043), as well as between low and high Internet use (*χ*^2^ = 7.5; df = 1; p = 0.006). For the establishment of contact with mental health professionals (*χ*^2^ = 2.1; df = 2; p = 0.358) and online communication with mental health professionals (*χ*^2^ = 0.8; df = 2; p = 0.682), differences in matters of user type were not statistically significant.

### Exchange of experience

**Figure 4:**
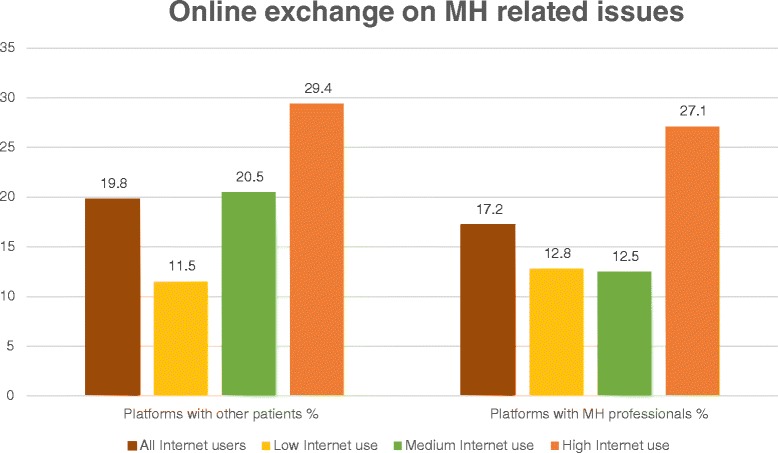
**Online exchange on mental health related issues by user type.** This figure illustrates the percentage of Internet users in the subsamples low, medium and high Internet use who use the above mentioned platforms. The percentage among all users is a reference value.

19.8% of the participants (53/268) used the Internet in order to exchange experiences with other patients (“peer-support”). 17.2% (46/268) used online platforms on which advice and information is supplied by mental health professionals (Figure 4).

**Figure 5:**
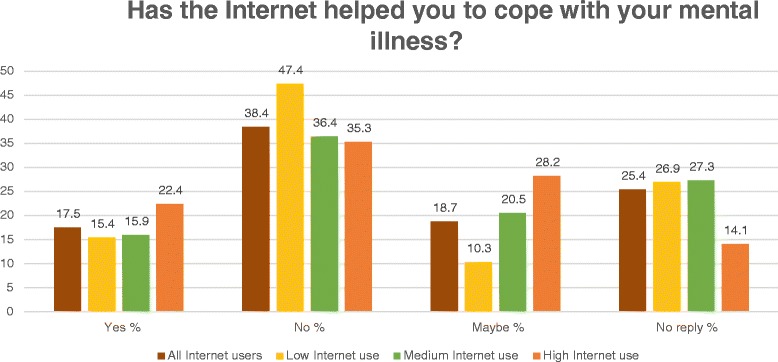
**The Internet’s effect on coping with mental illness by user type.** The answers to this question are displayed as a percentage of the subsamples low, medium and high Internet use. The percentage among all users is a reference value.

Regarding online exchange platforms with other patients, there was no statistically significant difference by user type (*χ*^2^ = 5.2; df = 2; p = 0.075), whereas the difference between user groups was statistically significant for the use of interactive platforms with mental health professionals (*χ*^2^ = 6.1; df = 2; p = 0.048). This is underlined by a significant difference between medium and high Internet use (*χ*^2^ = 5.4; df = 1; p = 0.020).

### The patients’ view on the Internet’s therapeutic options

#### Coping online

17.5% of the Internet users (47/268) believed that the Internet had helped them to cope with their mental illness, in contrast, 38.4% (103/268) were convinced that the Internet had not been helpful in contributing to their coping process. For 18.7% (50/268) the Internet may have been supportive in helping them to cope (Figure 5). There were no statistically significant group differences (*χ*^2^ = 8.6; df = 4; p = 0.072).

Arguments and counter-arguments concerning the Internet’s role in coping with a mental illness were given by 149 participants as open-ended answers and are displayed in Table 2. They illustrate the importance of psychoeducation by highlighting the “improved understanding of illness” as a major argument in favor of coping via Internet. However, this cannot replace personal interaction, which was the most important reason for our participants to reject the Internet’s possibilities for coping with a mental disorder.

**Table 2:** **Answers to open-ended questions about mental health related internet use**

**Arguments regarding the internet’s role in coping**	
	**Yes (n = 85)**
Improved understanding of illness	58
Exchange of experience and contact to others	19
Insight into illness	5
Availability of Internet services	3
	**No (n = 64)**
Lack of personal contact	38
Insufficiency and low quality of online information	16
Sufficient conventional therapy	10
**Arguments in favour of internet-based self-management (n = 53)**	
Psychoeducation	17
Proactivity and self-control	13
Availability and anonymity	9
Exchange with others	7
Ease of integration in daily routine	4
Information for family members	3

### Internet-based self-management

27.6% of the sample (74/268) liked to take part in web-based programs for the self-management of their mental disorder, while 60.1% (161/268) did not wish to do so. Differences between user groups were not statistically significant for this question (*χ*^2^ = 1.1; df = 2; p = 0.573).

Reasons why patients would wish for Internet-based self-management were given as a free text entry by 53 participants (Table 2) and consisted of 32.1% psychoeducation (17/53), 24.5% proactivity and self-control (13/53), 17.0% availability and anonymity (9/53), 13.2% exchange with others (7/53), 7.5% ease of integration in daily routine (4/53) and 5.7% information for family members (3/53).

### Differences in Internet usage depending on diagnosis and illness severity

In addition to the analysis mentioned above, Chi-square testing was carried out for the analysis of differences in reported Internet usage depending on a patient’s diagnosis. As affective disorders, schizophrenia and neurotic, stress-related and somatoform disorders (ICD-10 diagnoses F20-F48) covered 79.8% of all participants, the analysis was limited to these disorders.

Differences were neither statistically significant for reported Internet usage in general (*χ*^2^ = 2.7; df = 2; p = 0.254), nor for the usage of social media, such as social networks (*χ*^2^ = 1.0; df = 2; p = 0.614), forums (*χ*^2^ = 0.3; df = 2; p = 0.843), chat (*χ*^2^ = 0.8; df = 2; p = 0.687) and blogs (*χ*^2^ = 1.3; df = 2; p = 0.521).

Regarding the mental health related online contents used by patients, a statistically significant difference was found for “Online search for mental health professionals or services” (*χ*^2^ = 11.2; df = 2; p = 0.04), which was utilized by 13.3% (6/45) of Internet users with schizophrenia, by 46.0% (58/126) of Internet users suffering from depression and by 40.0% (20/50) of Internet users diagnosed with neurotic, stress-related and somatoform disorders. In the further testing for group differences, statistical significance was found in the comparison of the subgroups depression and schizophrenia (*χ*^2^ = 9.3; df = 1; p = 0.002), as well as for schizophrenia and neurotic disorders (*χ*^2^ = 9.5; df = 1; p = 0.002). As for the other mental health related online contents, “Information on mental disorders” (*χ*^2^ = 3.4; df = 2; p = 0.183), “Information on medication” (*χ*^2^ = 4.6; df = 2; p = 0.100), “Platforms with other patients” (*χ*^2^ = 0.8; df = 2; p = 0.670) and “Platforms with mental health professionals” (*χ*^2^ = 2.2; df = 2; p = 0.336), differences were not statistically significant. Similar results were shown for “Establishment of contact with mental health professionals via Internet” (*χ*^2^ = 1.8; df = 2; p = 0.406) and “Communication with mental health professionals via Internet” (*χ*^2^ = 0.0; df = 2; p = 0.988). In terms of “Coping” (*χ*^2^ = 3.3; df = 4; p = 0.506) and the “Internet-based self-management” (*χ*^2^ = 1.1; df = 2; p = 0.575), results were also shown to be not statistically significant.

In order to explore the relationship between illness severity (as assessed with GAF) and both general and mental health related Internet use, statistical testing was effectuated, but found no statistically significant cohesion (Table 3). This was also the case for an analysis using the CGI scores, which is included as Additional file 2.

**Table 3:** **Analysis of relationship between illness severity (GAF) and internet use**

	***χ*** ^**2**^	**df**	**p**
Internet use	3.6	7	0.827
Social networks	9.9	7	0.193
Forums	5.9	7	0.550
Chat	8.4	7	0.300
Blogs	2.0	7	0.959
Search for MH professionals or services	5.9	7	0.547
Information on mental disorders	7.5	7	0.377
Information on medication	6.3	7	0.513
Platforms with other patients	11.3	7	0.127
Platforms with MH professionals	5.7	7	0.578
Contact with MH professionals via Internet	2.6	7	0.919
Communication with MH professionals via Internet	2.6	6	0.854
Coping online	3.5	7	0.836
Internet-based self-management	7.7	7	0.364

## Discussion

### Information seeking is the key activity performed in mental health related Internet use

More than half of the participants look for information on mental disorders online and more than a third search for mental health services and professionals, using mainly search engines and online encyclopedias. The importance of Internet-based information seeking raises questions about the objectivity and quality of online information sources, which have been addressed not only by several studies [33-37], but also by participants in this survey (Table 2) – addressing the low quality of online information as an obstacle for coping via Internet.

According to Eysenbach et al. [38] the encounter of false online information depends on the quantity of incorrect information and the evaluation skills of the user. The latter requires specific training and the knowledge and employment of quality criteria [39].

In addition to information seeking, social media including social networks, chats and forums play an important role in both general and mental health related internet use. More than one in six patients use the option of sharing experience on the Internet with other patients or mental health professionals.

### Patients with high Internet use have a much stronger use of social media

Looking at results from previous surveys [4,29], we notice growing proportions of mental health related Internet use in general. In 2006 Powell and Clarke found that 20.5% of people with psychiatric history use the Internet for mental health related issues, while in 2008 Khazaal et al. identified 68.5% of mental health patients who looked for general health information online, without determining the proportion of patients looking for mental health related topics in particular. Our results show that 70.9% of Internet users search specifically for mental health related contents on the Web. This suggests a growth of mental health related Internet use over time, as Internet use in general continues to grow throughout the world [40].

Different types of users show statistically significant differences not only in matters of sociodemographic variables such as age, marital status, educational level and occupation, but also regarding the employment of interactive elements on the Web. We therefore assume that as Internet use continues to grow, the proportion of high Internet use and therefore strong usage of social media will rise as well. Data from the German general public support this hypothesis by showing an increase of social media usage in the last years [30]. For example, social networks were used by 15% of the German public in 2007, as opposed to 46% in 2013. In addition to this, the negative correlation between the reported frequency of Internet use and the users’ age illustrates a trend, which has also been addressed in statistics published by the European Commission [41]: Younger patients use the Internet more frequently – leading us to the conclusion that for future mental health patients interactive functions will continue to gain importance.

### Coping and self-management via Internet are seen with ambivalence by patients

The interpretation of the Internet’s role in the coping process is ambiguous. More than a third of the sample state that the Internet has not helped them with coping due to lack of personality and its questionable quality (Table 2). In contrast, almost the same number of patients believe that the Internet may have or has helped them to cope with their mental illness, because it offers options for mental health related communication and psychoeducation, a widely-used intervention [42]. Another advantage mentioned by patients is the high level of availability of online services.

In the current sample, more than half of the patients do not express a desire for online self-management tools. This opinion is shared by all users with no significant group difference and is illustrated as well by indicating conventional treatment options as sufficient for the coping process (Table 2). Somatic patients have been reported to show a similar attitude regarding the usage of online patient support groups [43]. It is thus important to devote attention to the persisting imbalance of supply and demand in Internet-based self-management, especially in the further development of online treatment options.

Reasons for the negative attitude towards online self-management programs were not specified in our survey. They have been explored in prior research, concluding that the negative perception of such programs mainly stems from the lack of immediate patient – therapist interaction [44,45]. In this context, patient education and counselling could be of great importance for the reduction of attitudinal barriers [46].

Nonetheless, more than a quarter of the patients declared interest in web-based self-help offers in the hope for psychoeducation, proactivity and self-control. Similar expectations have been found by Beattie et al. [44].

### Internet use does not vary between different types of psychiatric diagnoses

Neither the kind of a patient’s mental disorder, nor her/his degree of illness severity seems to influence the mental health related Internet use. Differences in the quantity and quality of reported Internet use were found to be not statistically significant in our data, with the exception of online search for mental health professionals. We therefore deduce that web-based therapy may be applicable for a wide variety of mental disorders. However, it is important to keep in mind that most programs are designed for patients with mild to moderate symptoms only and that online treatment is not yet accepted by all potential participants. Transdiagnostic approaches could be possible, particularly for disorders showing high proportions of comorbidity. Previous studies evaluating online therapy programs with transdiagnostic designs [47,48] have shown promising results, especially for the combined treatment of depression and anxiety disorders [10,49].

### Limitations

This study applies to a subset of psychiatric patients receiving care in a university hospital and therefore does not necessarily represent patients in primary care or without any treatment. It analyzes the Internet use reported by patients and did not measure actual Internet usage. Participants for this study were recruited from a single centre with a participation rate of 66%, resulting in a limited sample size. However, as patients from all treatment settings were included a broad spectrum of mental disorders and disease severity were covered.

Since the Internet follows a rapid evolution a prospective study design would be helpful for the evaluation of changes within patients over time.

## Conclusions

The importance of the Internet in mental health related contexts is unquestionable for psychiatric patients regardless of their diagnosis. Information seeking is the predominant Internet function in mental health related Internet use, whereas social media is of secondary relevance. Patients with high Internet use show the most frequent application of such. The possibilities for coping and self-management on the Internet are seen with ambivalence by the participants of this survey.

The results reported in this current study illustrate that Internet access is readily available for the majority of mental health patients, but the utilization of social media remains unfamiliar for many of them. This should be taken into account when developing Internet-based therapy or self-management programs for patients suffering from psychiatric disorders.

## Additional files

Additional file 1:
**Sample questions.**
Additional file 2:
**Analysis of relationship between illness severity (CGI) and Internet use.**

## References

[1] McDaid D, Park A: **Online Health: Untangling The Web.** [http://www.bupa.com/media/44806/online_20health_20-_20untangling_20the_20web.pdf]

[2] Andreassen HK, Bujnowska-Fedak MM, Chronaki CE, Dumitru RC, Pudule I, Santana S, Voss H, Wynn R (2007). European citizens’ use of E-health services: a study of seven countries. BMC Public Health.

[3] Wangberg S, Andreassen H, Kummervold P, Wynn R, Sorensen T (2009). Use of the internet for health purposes: trends in Norway 2000–2010. Scand J Caring Sci.

[4] Powell J, Clarke A (2006). Internet information-seeking in mental health: population survey. Br J Psychiatry.

[5] Styra R (2004). The Internet’s impact on the practice of psychiatry. Can J Psychiatry.

[6] Oh E, Jorm AF, Wright A (2009). Perceived helpfulness of websites for mental health information. Soc Psychiat Epidemiol.

[7] Klein JP, Berger T, Schroder J, Spath C, Meyer B, Caspar F, Lutz W, Greiner W, Hautzinger M, Rose M, Grafe V, Hohagen F, Andersson G, Vettorazzi E, Moritz S (2013). The EVIDENT-trial: protocol and rationale of a multicenter randomized controlled trial testing the effectiveness of an online-based psychological intervention. BMC Psychiatry.

[8] Buntrock C, Ebert DD, Lehr D, Cuijpers P, Riper H, Smit F, Berking M (2014). Evaluating the efficacy and cost-effectiveness of web-based indicated prevention of major depression: design of a randomised controlled trial. BMC Psychiatry.

[9] Kordy H, Backenstrass M, Hüsing J, Wolf M, Aulich K, Bürgy M, Puschner B, Rummel-Kluge C, Vedder H: **Supportive monitoring and disease management through the internet: An internet-delivered intervention strategy for recurrent depression.** *Contemp Clin Trials* 2013, **36**(2):327–329. 10.1016/j.cct.2013.08.005 23974036

[10] Titov N, Dear BF, Schwencke G, Andrews G, Johnston L, Craske MG, McEvoy P (2011). Transdiagnostic internet treatment for anxiety and depression: a randomised controlled trial. Behav Res Ther.

[11] Hedman E, Ljótsson B, Lindefors N (2012). Cognitive behavior therapy via the Internet: a systematic review of applications, clinical efficacy and cost–effectiveness. Expert Rev Pharmacoeconomics Outcomes Res.

[12] Andersson G, Bergstrom J, Hollandare F, Carlbring P, Kaldo V, Ekselius L (2005). Internet-based self-help for depression: randomised controlled trial. Br J Psychiatry.

[13] Perini S, Titov N, Andrews G (2009). Clinician-assisted Internet-based treatment is effective for depression: randomized controlled trial. Aust NZ J Psychiatry.

[14] Kessler D, Lewis G, Kaur S, Wiles N, King M, Weich S, Sharp DJ, Araya R, Hollinghurst S, Peters TJ (2009). Therapist-delivered internet psychotherapy for depression in primary care: a randomised controlled trial. Lancet.

[15] Andersson G, Cuijpers P (2009). Internet-based and other computerized psychological treatments for adult depression: a meta-analysis. Cogn Behav Ther.

[16] Cuijpers P, Donker T, Johansson R, Mohr DC, van Straten A, Andersson G (2011). Self-guided psychological treatment for depressive symptoms: a meta-analysis. PLoS One.

[17] Johansson R, Andersson G (2012). Internet-based psychological treatments for depression. Expert Rev Neurotherapeutics.

[18] Moritz S, Schilling L, Hauschildt M, Schroder J, Treszl A (2012). A randomized controlled trial of internet-based therapy in depression. Behav Res Ther.

[19] Williams AD, Andrews G, Andersson G (2013). The Effectiveness of Internet Cognitive Behavioural Therapy (iCBT) for depression in primary care: a quality assurance study. PLoS One.

[20] Hedman E, Ljótsson B, Kaldo V, Hesser H, El Alaoui S, Kraepelien M, Andersson E, Rück C, Svanborg C, Andersson G, Lindefors N: **Effectiveness of Internet-based cognitive behaviour therapy for depression in routine psychiatric care.** *J Affect Dis* 2013, **155:**49–58. 10.1016/j.jad.2013.10.023 24238951

[21] Proudfoot J, Clarke J, Birch M, Whitton AE, Parker G, Manicavasagar V, Harrison V, Christensen H, Hadzi-Pavlovic D (2013). Impact of a mobile phone and web program on symptom and functional outcomes for people with mild-to-moderate depression, anxiety and stress: a randomised controlled trial. BMC Psychiatry.

[22] Andrews G, Cuijpers P, Craske MG, McEvoy P, Titov N, Baune BT (2010). Computer therapy for the anxiety and depressive disorders is effective, acceptable and practical health care: a meta-analysis. PLoS ONE.

[23] Mewton L, Wong N, Andrews G (2012). The effectiveness of internet cognitive behavioural therapy for generalized anxiety disorder in clinical practice. Depress Anxiety.

[24] Stott R, Wild J, Grey N, Liness S, Warnock-Parkes E, Commins S, Readings J, Bremner G, Woodward E, Ehlers A, Clark DM (2013). Internet-delivered cognitive therapy for social anxiety disorder: a development pilot series. Behav Cogn Psychother.

[25] Bauer S, Kordy H (2008). E-Mental-Health: Neue Medien in der psychosozialen Versorgung.

[26] Alonso J, Angermeyer MC, Bernert S, Bruffaerts R, Brugha TS, Bryson H, Girolamo G de, Graaf R, Demyttenaere K, Gasquet I, Haro JM, Katz SJ, Kessler RC, Kovess V, Lépine JP, Ormel J, Polidori G, Russo LJ, Vilagut G, Almansa J, Arbabzadeh-Bouchez S, Autonell J, Bernal M, Buist-Bouwman MA, Codony M, Domingo-Salvany A, Ferrer M, Joo SS, Martínez-Alonso M, Matschinger H *et al.*: **Prevalence of mental disorders in Europe: results from the European Study of the Epidemiology of Mental Disorders (ESEMeD) project.** *Acta Psychiatr Scand Suppl* 2004:21–27 10.1111/j.1600-0047.2004.00327.x 15128384

[27] Fryers T, Melzer D, Jenkins R (2003). Social inequalities and the common mental disorders: a systematic review of the evidence. Soc Psychiatry Psychiatr Epidemiol.

[28] Wöller A: **Internetnutzung von psychiatrischen Patienten.** *PhD thesis.* University of Munich, Germany, Department of Psychiatry and Psychotherapy. Munich, Germany; 2005.

[29] Khazaal Y, Chatton A, Cochand S, Hoch A, Khankarli MB, Khan R, Zullino DF (2008). Internet use by patients with psychiatric disorders in search for general and medical informations. Psychiatr Q.

[30] van Eimeren B, Frees B: *Rasanter Anstieg des Internetkonsums - Onliner fast drei Stunden täglich im Netz. Ergebnisse der ARD/ZDF-Onlinestudie 2013*.

[31] Endicott J, Spitzer RL, Fleiss JL, Cohen J (1976). The global assessment scale. A procedure for measuring overall severity of psychiatric disturbance. Arch Gen Psychiatry.

[32] Guy W: **ECDEU assessment manual for psychopharmacology. Revised (DHEW Publ No ADM 76–338).** [https://archive.org/details/ecdeuassessmentm1933guyw]

[33] Griffiths KM, Christensen H (2000). Quality of web based information on treatment of depression: cross sectional survey. BMJ.

[34] Khazaal Y, Chatton A, Cochand S, Zullino D (2008). Quality of Web-based information on cocaine addiction. Patient Educ Couns.

[35] Morel V, Chatton A, Cochand S, Zullino D, Khazaal Y (2008). Quality of web-based information on bipolar disorder. J Affect Disord.

[36] Reavley NJ, Jorm AF (2011). The quality of mental disorder information websites: a review. Patient Educ Couns.

[37] Reavley NJ, Mackinnon AJ, Morgan AJ, Alvarez-Jimenez M, Hetrick SE, Killackey E, Nelson B, Purcell R, Yap MBH, Jorm AF (2012). Quality of information sources about mental disorders: a comparison of Wikipedia with centrally controlled web and printed sources. Psychol Med.

[38] Eysenbach G, Powell J, Kuss O, Sa E (2002). Empirical studies assessing the quality of health information for consumers on the world wide web: a systematic review. JAMA.

[39] Cline RJ, Haynes KM (2001). Consumer health information seeking on the Internet: the state of the art. Health Educ Res.

[40] International Telecommunication Union: **The world in 2013: ICT Facts and Figures.** [http://www.itu.int/en/ITU-D/Statistics/Documents/facts/ICTFactsFigures2013-e.pdf]

[41] European Commission: **Internet use statistics.** [http://epp.eurostat.ec.europa.eu/statistics_explained/index.php/Internet_use_statistics_-_individuals#Internet_use_by_individuals]

[42] Rummel-Kluge C, Kluge M, Kissling W (2013). Frequency and relevance of psychoeducation in psychiatric diagnoses: results of two surveys five years apart in German-speaking European countries. BMC Psychiatry.

[43] Van Uden-Kraan CF, Drossaert CH, Taal E, Smit WM, Moens HJ, Bernelot, Siesling S, Seydel ER, Van de Laar MAFJ (2009). Health-related Internet use by patients with somatic diseases: frequency of use and characteristics of users. Inform Health Soc Care.

[44] Beattie A, Shaw A, Kaur S, Kessler D (2009). Primary-care patients’ expectations and experiences of online cognitive behavioural therapy for depression: a qualitative study. Health Expect.

[45] Wilhelmsen M, Lillevoll K, Risør MB, Høifødt R, Johansen M, Waterloo K, Eisemann M, Kolstrup N (2013). Motivation to persist with internet-based cognitive behavioural treatment using blended care: a qualitative study. BMC Psychiatry.

[46] Waller R, Gilbody S (2009). Barriers to the uptake of computerized cognitive behavioural therapy: a systematic review of the quantitative and qualitative evidence. Psychol Med.

[47] Ebert D, Tarnowski T, Gollwitzer M, Sieland B, Berking M (2013). A transdiagnostic internet-based maintenance treatment enhances the stability of outcome after inpatient cognitive behavioral therapy: a randomized controlled trial. Psychother Psychosom.

[48] Ebert DD, Gollwitzer M, Riper H, Cuijpers P, Baumeister H, Berking M (2013). For Whom Does It Work? Moderators of outcome on the effect of a transdiagnostic internet-based maintenance treatment after inpatient psychotherapy: randomized controlled trial. J Med Internet Res.

[49] Dear BF, Titov N, Schwencke G, Andrews G, Johnston L, Craske MG, McEvoy P (2011). An open trial of a brief transdiagnostic internet treatment for anxiety and depression. Behav Res Ther.

