# Supplementary material for: Mental health related Internet use among psychiatric patients: a cross-sectional analysis
Source: BMC Psychiatry. 2014 Dec 24;14:368. doi: 10.1186/s12888-014-0368-7 (PMC4299476; doi:10.1186/s12888-014-0368-7)
Supplement: Additional file 1: — Sample questions. [file 12888_2014_368_MOESM1_ESM.pdf]

Additional file 1: Sample questions

Question 2.12: Has the Internet helped you with coping with your mental illness?

- ○ Yes, because …………………………………………………………………………………..
- ○ Maybe, because………………………………………………………………………………
- ○ No, because……………………………………………………………………………………

Question 2.15: Which mental health related online contents are you interested in? (Multiple answers possible)

- ○ Information on mental disorders
- ○ Information on medication (e.g. antidepressants, neuroleptics)
- ○ Search for mental health professionals or services
- ○ Exchange of experience with other patients
- ○ Platforms with mental health professionals
- ○ Other contents: ……………………………………………………………………………….
