# Supplementary material for: Mental health related Internet use among psychiatric patients: a cross-sectional analysis
Source: BMC Psychiatry. 2014 Dec 24;14:368. doi: 10.1186/s12888-014-0368-7 (PMC4299476; doi:10.1186/s12888-014-0368-7)
Supplement: Additional file 2: — Analysis of relationship between illness severity (CGI) and Internet use. [file 12888_2014_368_MOESM2_ESM.pdf]

Additional file 2: Analysis of relationship between illness severity (CGI) and Internet use

CGI

	χ^2	df	p
Internet use	4.6	5	0.472
Social networks	3.2	5	0.665
Forums	1.0	5	0.963
Chat	1.4	5	0.924
Blogs	1.4	5	0.923
Search for MH professionals or services	4.6	4	0.334
Information on mental disorders	4.6	4	0.330
Information on medication	1.2	4	0.876
Platforms with other patients	8.0	4	0.092
Platforms with MH professionals	2.4	4	0.668
Contact with MH professionals via Internet	11.0	5	0.052
Communication with MH professionals via Internet	1.1	4	0.898
Coping online	4.9	5	0.428
Internet-based self-management	4.6	5	0.467
